# Flavivirus-based bivalent nanoparticle vaccines induce neutralizing antibodies and Th1 responses against flavivirus and coupling antigens

**DOI:** 10.1016/j.isci.2025.113659

**Published:** 2025-10-04

**Authors:** Koga Ii, Fumitaka Sato, Yu Hatakeyama, Hidehiko Suzuki, Takafumi Noguchi, Kotaro Ishida, Masashi Arakawa, Kazumasa Nakamura, Ryuta Iwatsuki, Cong Thanh Nguyen, Akiho Yoshida, Nobuyuki Tanaka, Ikuo Tsunoda, Hirotaka Ebina, Eiji Morita

**Affiliations:** 1 Department of Biochemistry and Molecular Biology, Faculty of Agriculture and Life Science, Hirosaki University, 3 Bunkyo-cho, Hirosaki 036-8561, Aomori, Japan; 2 Department of Microbiology, Kindai University Faculty of Medicine, 377-2 Ōnohigashi, Ōsakasayama, Osaka 589-0014, Japan; 3 The Research Foundation for Microbial Diseases of Osaka University, 3-1 Yamadaoka, Suita Osaka 565-0871, Japan; 4 Virus Vaccine Group, BIKEN Innovative Vaccine Research Alliance Laboratories, Institute for Open and Transdisciplinary Research Initiatives, 3-1 Yamadaoka, Suita Osaka 565-0871, Japan; 5 Research Institute for Microbial Diseases, Osaka University, 3-1 Yamadaoka, Suita Osaka 565-0871, Japan; 6 Center for Advanced Modalities and DDS (CAMaD), Osaka University, 3-1 Yamadaoka, Suita Osaka 565-0871, Japan; 7 Center for Infectious Disease Education and Research (CiDER), Osaka University, 3-1 Yamadaoka, Suita Osaka 565-0871, Japan; 8 Division of Tumor Immunobiology, Miyagi Cancer Center Research Institute, 47-1 Nodayama, Medeshimashiode, Natori 981-1293, Miyagi, Japan; 9 Division of Tumor Immunobiology, Tohoku University Graduate School of Medicine, 2-1 Seiryo-machi, Aoba-ku, Sendai 980-8575, Miyagi, Japan

**Keywords:** natural sciences, Biological sciences, Immunology

## Abstract

Effective vaccines against flaviviruses, including Japanese encephalitis virus (JEV), are widely used. We developed antigen-loaded nanoparticle vaccines, using JEV virus-like particles (JEV-VLPs) as carriers. The severe acute respiratory syndrome coronavirus 2 (SARS-CoV-2) spike receptor-binding domain (S-RBD) was covalently coupled to specific peptide insertion sites on the JEV envelope protein. Mice immunized with these coupled particles (JEprME-S-RBD) produced high levels of neutralizing antibodies against both JEV and SARS-CoV-2, with broad activity against multiple SARS-CoV-2 variants. These mice also had a larger number of interferon (IFN)-γ-producing cells with higher levels of anti-S-RBD IgG2a production than mice receiving the JEV-VLP and S-RBD without coupling. These results suggested the induction of T helper (Th) 1 immune responses. We also constructed JEV nanoparticles coupled with human parvovirus B19 RBD, which efficiently induced neutralizing antibodies against both JEV and parvovirus. Therefore, flaviviral nanoparticles can serve as versatile multivalent vaccine platforms by incorporating neutralizing epitopes from different pathogens.

## Highlights

• JEV nanoparticles were coupled to the SARS-CoV-2 S-RBD antigen
• Immunization with nanoparticles induced neutralizing antibodies against both antigens
• Immunization with nanoparticles also induced Th1 immune responses
• JEV nanoparticles have potential as carriers for multivalent vaccines

## Graphical abstract

**Figure:**
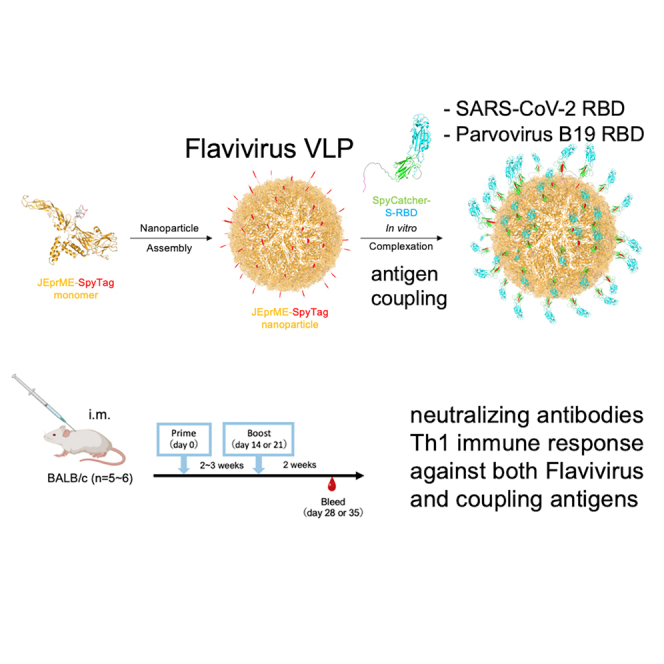

## Introduction

Protein nanoparticles are self-assembled particles with diameters ranging from 20 to 100 nm, forming regular polyhedral spherical structures with thermodynamic stability, and have drawn attention as carriers for vaccine antigens.^1^ The nanoparticles have also been shown to be efficiently recognized by the immune system and can sustain antigenicity for an extended duration, which can reduce vaccine dosages and potentially diminish the requirement for adjuvants. Various nanoparticle vaccines against severe acute respiratory syndrome coronavirus (SARS-CoV)-2 and other viruses have been developed, which are composed of scaffold core proteins, such as I53-50,^2^^,^^3^ ferritin,^4^^,^^5^^,^^6^ I3-01,^7^^,^^8^ lumazine synthase,^9^ and bacteriophage AP205 capsid-like particles.^10^ These anti-viral nanoparticle vaccines have been shown to induce robust anti-viral humoral immune (antibody) responses and cellular immune (T cell) responses, which play central roles in preventing viral infections.^11^ However, certain antiviral nanoparticle vaccines have been reported to stimulate immune responses not only against target antigens but also against the scaffold core proteins of the nanoparticles. This has raised concerns that immune responses targeting core proteins might lead to faster clearance of vaccines or cause unforeseen adverse effects.^12^

Flaviviruses (orthoflavivirus) are arthropod-borne pathogens responsible for substantial global mortality and morbidity,^13^ including JEV, dengue virus (DENV), yellow fever virus (YFV), Zika virus (ZIKV), West Nile virus (WNV), and tick-borne encephalitis virus (TBEV).^13^ Effective vaccines for YFV, JEV, and TBEV have already been developed and are widely utilized.^14^^,^^15^^,^^16^ Flaviviruses are enveloped viruses with an 11 kb single-stranded, positive-sense RNA genome.^17^ The viral genome contains a single open reading frame that is cleaved into three structural (capsid [C], pre-membrane [prM], and envelope [E]) and seven nonstructural proteins (NSPs). The prM and E structural proteins form an envelope that encapsulates the nucleocapsid cores. During maturation, prM is cleaved by the Golgi enzyme furin to form M.^18^ Within immature intracellular particles, the prM protein forms heterodimers with the E protein (prME) on the surface of the viral particles. The E protein comprises three domains, with the receptor-binding domain (RBD) located in domain III, where numerous neutralizing antibody epitopes are localized. The expression of prM and E proteins in cells leads to the production of virus-like particles (VLPs) lacking nucleocapsids.^19^ These VLPs are referred to as sub-viral particles, have a mean diameter of 30–50 nm, and are composed of M and E proteins anchored to the outer surface of the virion.^20^ Natural mature VLPs, which contain multiple copies of the M and E proteins embedded in the lipid envelope, exhibit properties similar to those of infectious virions.^21^ The assembly and maturation of these particles are regulated by host enzymes such as signal peptidases and furin proteases.^21^ Consequently, VLPs can exist in different maturation states during morphogenesis: mature, immature, and partially mature. Mature VLPs comprise M and E protein dimers, whereas immature VLPs comprise prM and E protein trimers. Immature VLPs lack fusion activity and often display spiky surface morphology. Additionally, under acidic conditions (pH < 6), mature VLPs undergo an irreversible conformational change from a smooth to a spiky form due to the formation of E protein trimers.^21^ Immunization with these VLPs elicits the production of neutralizing antibodies.^22^ Several insertion sites for foreign epitopes have been identified on the surface of JEV particles, particularly on the E protein.^23^

SARS-CoV-2, the causative agent of coronavirus disease (COVID-19), was first reported in December 2019 in Wuhan, China.^24^ Since then, SARS-CoV-2 has spread worldwide, and the World Health Organization declared it a pandemic in March 2020. Although various vaccine modalities have been developed against COVID-19, there is still a need to develop novel vaccines that exhibit higher efficacy and fewer adverse effects to address the newly emerging variants. SARS-CoV-2 is an enveloped virus with a single-stranded positive-sense RNA genome that belongs to the genus *Betacoronavirus*, family Coronaviridae.^24^ Viral RNA encodes four structural proteins: spike (S), E, membrane, and nucleocapsid proteins, 16 NSPs, and nine accessory proteins.^25^ The S protein consists of an ectodomain (comprising S1 and S2 subunits), a transmembrane domain, and an intracellular domain. Similar to SARS-CoV, SARS-CoV-2 binds to human angiotensin-converting enzyme through the RBD within the S1 subunit, facilitating membrane fusion via the S2 subunit for genome release into the cytoplasm.^26^ Thus, the neutralizing epitopes predominantly target S-RBD during infection.

Human parvovirus B19 (B19V) causes erythema infectiousum, commonly known as “fifth disease,” characterized by a red rash on the cheeks and a high fever in infected children.^27^ During pregnancy, B19V can cross the placenta, potentially resulting in severe fetal complications such as hydrops fetalis and anemia, as well as miscarriage in the most severe cases. No effective vaccines against B19V have been developed (Chisaka et al. 2003). The B19V particle comprises two structural proteins, VP1 and VP2, with an RBD and neutralizing epitopes located within the 5–80 amino acid region of the VP1 protein.^28^ Previously, we demonstrated that B19V-RBD conjugated with ferritin or I3-05, which are self-assembling protein nanoparticles, can serve as promising vaccine candidates by efficiently inducing the production of neutralizing antibodies.^29^

**Figure 1:**
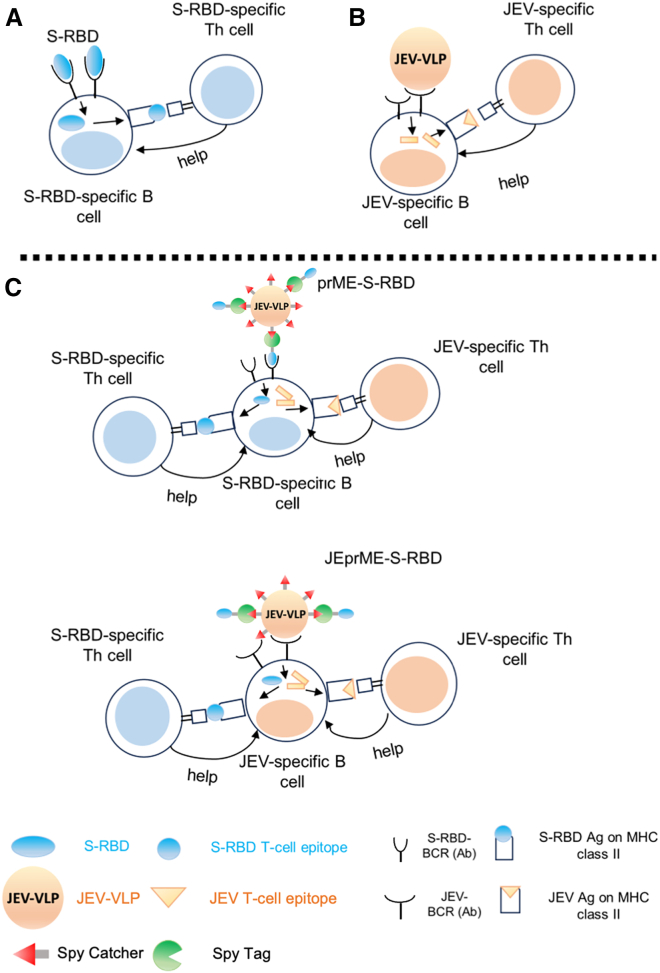
Working hypothesis of linked recognition of coronavirus spike-receptor binding domain and Japanese encephalitis virus antigens, enhancing antibody responses to two viruses (A) When the S-RBD vaccine alone was given for immunization, S-RBD-specific B cells captured S-RBD by B cell receptors (BCRs) and presented S-RBD antigens to S-RBD-specific helper T (Th) cells on major histocompatibility complex (MHC) class II molecules; S-RBD-specific Th cells in turn would produce cytokines, helping antibody production and isotype switching of B cells. (B) When JEV virus-like particle (VLP) vaccine alone was given, JEV-specific Th cells would help JEV-specific antibody production. (A and B) When the mixture of S-RBD and JEV-VLP vaccines was administered, S-RBD-specific Th cells or JEV-specific Th cells could be induced independently by S-RBD-specific B cells or JEV-specific B cells, respectively. Here, S-RBD-specific Th cells would help S-RBD-specific B cells’ antibody production; JEV-specific Th cells would help JEV-specific B cells’ antibody production. (C) We constructed a novel nanoparticle vaccine, JEprME-S-RBD, containing S-RBD linked to the E protein of JEV-VLPs. When JEprME-S-RBD nanoparticles were given for immunization, both S-RBD-specific and JEV-specific B cells captured and internalized the nanoparticles, and then presented S-RBD and JEV antigens to either S-RBD-specific or JEV-specific Th cells, respectively, due to the linked recognition. Here, in JEprME-S-RBD immunizations, S-RBD-specific B cells can receive the T cell help from not only S-RBD-specific Th cells but also JEV-specific Th cells; similarly, JEV-specific B cells can receive the help from both JEV-specific and S-RBD-specific Th cells.

In T cell-dependent antibody responses, helper T (Th) cells are activated by antigen fragments also recognized by B cells, which in turn are optimally activated with the assistance of these Th cells. This is called “linked recognition.” The epitopes recognized by T cell receptors often differ from those recognized by B cell receptors (i.e., antibodies).^30^ Nanoparticles are internalized by B cells via endocytosis.^31^ In theory, when the antigen of interest is linked to nanoparticle core proteins, antigen-loaded nanoparticle vaccine immunization can induce Th cells specific to the nanoparticle core proteins, which may promote antigen-specific antibody responses regardless of the choice of nanoparticle core proteins. Viruses contain multiple T cell and B cell epitopes and linked recognition can occur because T cell epitopes are physically associated with B cell epitope antigens in the virions. Thus, we hypothesized that using VLPs as nanoparticle vaccine core proteins, immunization with VLPs containing the antigen of interest could result in the induction of linked recognition and VLP-specific Th cells that may optimize the antibody response to the antigen of interest (Figure 1).

In this study, we constructed JEV-VLP-containing SARS-CoV-2 S-RBD as a protein nanoparticle vaccine. We demonstrated that the linked recognition of flavivirus and coronavirus antigens results in the induction of both flavivirus- and coronavirus-specific Th cells, each of which enhances antibody responses to both flaviviruses and coronaviruses. We also demonstrated that the coupled antigen could be replaced by another viral antigen, the B19V RBD, because the JEV-VLP-containing B19V RBD efficiently induced neutralizing antibodies against both JEV and B19V.

## Results

### Development of JEprME nanoparticle containing SpyTag peptide

The JEV E protein contains a single N-glycosylation site (N154) at the 154th asparagine residue; in the related flavivirus DENV, besides N154, there is an additional N-glycosylation site (N67). The D67N mutation in JEV-E has been shown to cause excessive glycosylation, leading to increased expression, solubility, and secretion levels of subviral particles by more than 10 times compared to the wild-type.^32^ Using JEV-E (D67N), we tested potential peptide insertion sites based on the structural information of JEV-E (PDB: 3P54)^33^ for presentation on the surface of VLP nanoparticles. Based on the structure of mature JEV particles, we inserted the peptide into surface-exposed loops or, alternatively, replaced entire loops or strands within the β-sheet structure.

**Figure 2:**
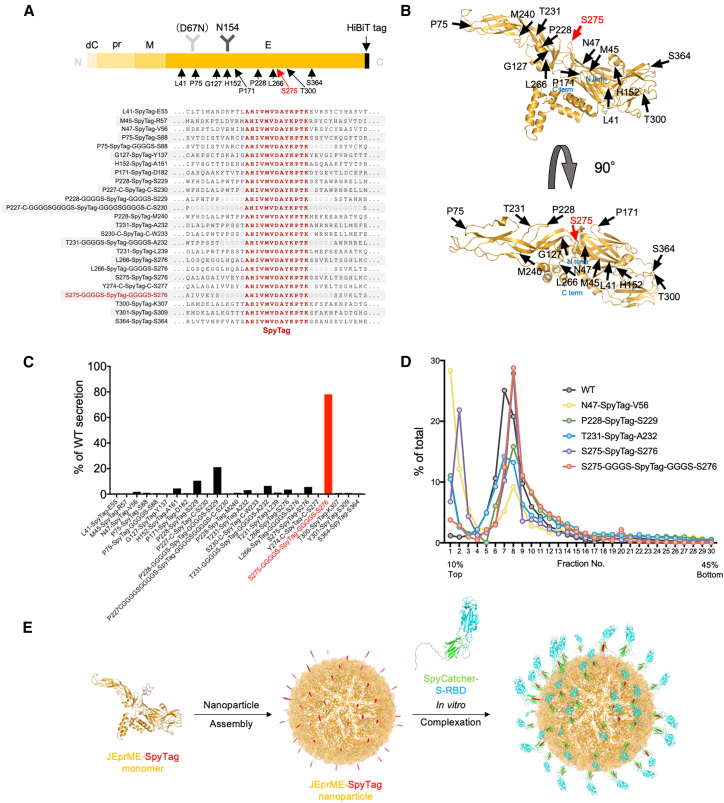
Design of JEprME-S-RBD nanoparticles and identification of SpyTag insertion sites (A) Primary structures of JEprME-HiBiT and SpyTag insertion mutants. The SpyTag sequence (in red) and GS linker sequence (in gray) are indicated. dC: C-terminal 22 amino acids of the capsid. (B) Structure of the JE-prME monomer (Protein DataBank [PDB] 5WSN) and peptide insertion sites. Arrows and numbers indicate the representative 14 SpyTag insertion sites. The two structures represent a 90° rotation. (C) Detection of HiBiT-dependent NanoLuc luciferase activity (HiBiT activity) in the culture supernatant of HEK293T cells expressing SpyTag insertion mutants of JEprME-HiBiT. HiBiT activity in the culture supernatant was measured 48 h after transfection. The *y* axis represents the relative secretion level compared with JE-prME wild-type (WT). (D) Sucrose density gradient ultracentrifugation (10%–45% w/v) analysis of SpyTag insertion JE-prME mutants. Each construct was transiently expressed in HEK293T cells. After 48-h cultivation, HiBiT activity in each fraction of the culture supernatant was measured. (E) Schematic diagram of severe acute respiratory syndrome coronavirus (SARS-CoV)-2 S-RBD binding to JE-prME particles via SpyTag-SpyCatcher interaction. JE-prME, orange; SpyTag, red; S-RBD, cyan; SpyCatcher, green; His tag, pink; and GS linker, gray.

The SpyTag-SpyCatcher system^34^ was used to couple the SARS-CoV-2 S-RBD to the JEV virus-like particles (JEV-VLPs). The FbaB protein, derived from *Streptococcus pyogenes*, contains a domain in which spontaneous covalent bonds are formed between lysine and aspartic acid residues. This domain can be split into two protein modules (SpyTag and SpyCatcher) that form specific covalent bonds when each module interacts, providing a system for sequence-specific covalent binding induction. In addition, SpyTag, a short peptide tag consisting of 13 amino acids, has the advantage of having minimal influence on protein folding. We generated 25 SpyTag insertion or substitution variants (Figures 2A and 2B) and assessed the extracellular release of prME from 293T cells (Figure 2C). The C-terminus of prME was fused with the HiBiT tag, which is a component of the split NanoLuc luciferase system, facilitating sensitive detection of secreted prME.^32^ We detected nine variants (N47-SpyTag-V56, H152-SpyTag-A161, T231-SpyTag-L239, P228-SpyTag-S229, P228-GGGGS-SpyTag-GGGGS-S229, T231-GGGGS-SpyTag-GGGGS-A232, S275-SpyTag-S276, L266-SpyTag-GGGGS-S276, and S275-GGGGS-SpyTag-GGGGS-S276) with significant increases in the HiBiT tag-dependent NanoLuc luciferase activity (HiBiT activity) in the culture supernatants. The S275-GGGGS-SpyTag-GGGGS-S276 mutant (JEprME-SpyTag) exhibited the highest HiBiT activity. We fractionated the culture supernatants using sucrose density gradient ultracentrifugation and found peaks corresponding to assembled VLPs (Figure 2D). These results indicated that JEprME-SpyTag nanoparticles were secreted into the culture supernatants. Thus, we coupled SARS-CoV-2 S-RBD (SpyCatcher-RBD) fused with SpyCatcher to JEprME-SpyTag nanoparticles to produce JEV nanoparticles with SARS-CoV-2 S-RBD on the surface (Figure 2E).

### Purification of JEprME-SpyTag nanoparticles and binding with SpyCatcher-S-RBD

**Figure 3:**
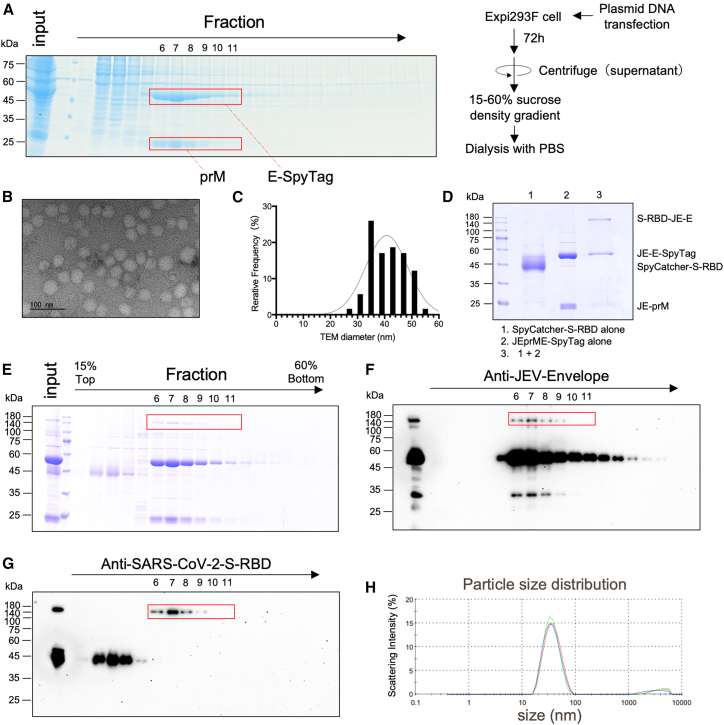
Purification of JEprME-SpyTag nanoparticles and binding with SpyCatcher-SARS-CoV-2 S-RBD (A) Purification of JEprME-SpyTag particles by sucrose density gradient ultracentrifugation and simplified purification protocol. Culture supernatants of Expi293F cells transfected with plasmids were collected, fractionated by 15%–60% (w/v) sucrose density gradient ultracentrifugation, and separated by sodium dodecyl sulfate polyacrylamide gel electrophoresis (SDS-PAGE) followed by Coomassie brilliant blue (CBB) staining. The red boxes in fractions 6–11 indicate E-SpyTag and prM. (B) Transmission electron microscopy (TEM) image of negatively stained purified JEprME-SpyTag particles. Fractions 6–11 from the analysis in (A) were collected and dialyzed in PBS. Concentrated particles were observed by TEM. Scale bars: 100 nm. (C) Histogram showing particle size distributions. Particle size was measured based on the negatively stained images obtained in (B). The number of particles measured was 123. (D) Reducing SDS-PAGE showing the binding of JE-prME particles to S-RBD. Lane 1: purified SpyCatcher-RBD (40 kDa) alone, lane 2: JEprME-SpyTag monomer (E-SpyTag: 55 kDa, prM: 21 kDa) alone, and lane 3: JEprME-S-RBD nanoparticles after overnight incubation at 4°C. Reducing SDS-PAGE showing the purification process of JEprME-S-RBD nanoparticles. After binding, the nanoparticles were fractionated again by 15%–60% (w/v) sucrose density gradient ultracentrifugation to remove unbound SpyCatcher-RBD. (E–G) JEprME-S-RBD nanoparticles were detected using CBB staining (E), anti-JEV-E antibody (F), and anti-SARS-CoV-2 S-RBD antibody (G). The red box indicates the covalently bound JEprME-S-RBD. (H) Results of dynamic light scattering (DLS) analysis for purified JEprME-S-RBD nanoparticles.

The JEprME-SpyTag expression vector was transfected into Expi293F cells and the culture supernatants were collected after 72 h of cultivation. After the culture supernatants were fractionated into 30 fractions using 15%–60% (w/v) sucrose density gradient ultracentrifugation, fractions 6–11 containing prME were collected, dialyzed overnight in phosphate-buffered saline (PBS), and then concentrated using ultrafiltration to obtain JEprME-SpyTag nanoparticles (Figure 3A). Bands corresponding to prM and E-SpyTag were detected in collected fractions. We conducted electron microscopic image analyses of negatively stained nanoparticles and found relatively uniform particle formation of the JEprME-SpyTag nanoparticles (Figures 3B and 3C). Subsequently, a ligand was fused to SpyCatcher at the N-terminus of SARS-CoV-2-RBD (Wuhan strain) (Figure S1) and reacted with the nanoparticles. Upon electrophoresis of the reaction products, an additional band of around 180 kDa was detected besides the E (55 kDa) and prM (24 kDa) proteins (Figure 3D); upon fractionating the reaction mixture by sucrose density gradient ultracentrifugation again, this band was detected in the same fractions as the prM and E proteins (Figure 3E). We detected the formation of a covalent isopeptide bond between SpyCatcher and SpyTag using antibodies against JEV-E and SARS-CoV-2 S-RBDs (Figures 2F and 2G). The band intensity in the SDS-PAGE analysis revealed that the ratio of S-RBD-bound prME to unbound prME in fractions 6–11 was approximately 1:32. Dynamic light scattering analysis of purified JEprME-S-RBD confirmed the presence of a single nanoparticle population with an average diameter of 37.39 ± 1.30 nm (Figure 3H). Because these fractions did not contain free ligands, they were utilized as ligand-bound nanoparticles (JEprME-S-RBD) in the subsequent immunological experiments.

### Immunization of mice with JEprME-S-RBD nanoparticles induces high levels of antigen-specific neutralizing antibodies against both JEV and SARS-CoV-2 RBD

**Figure 4:**
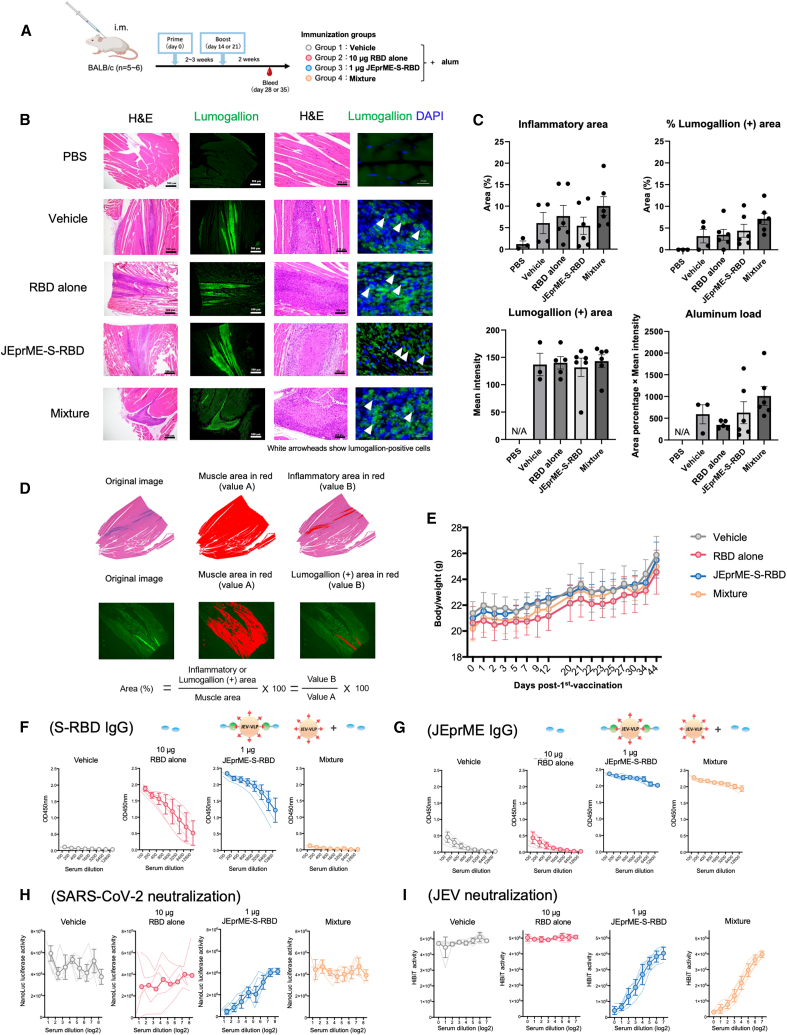
Induction of antigen-specific neutralizing antibodies against both JEV and SARS-CoV-2 by immunization with JEprME-S-RBD nanoparticles in mice (A) BALB/c mice (*n* = 4–6 per group) were injected in the quadriceps muscle with vehicle (group 1), 10 μg of soluble S-RBD (roup 2), 1 μg of JEprME-S-RBD particles containing S-RBD (group 3), and non-binding mixture (JEprME-SpyTag and soluble S-RBD) (group 4) with alum adjuvant at 2-week intervals. In all groups, the inoculum was given in alum adjuvant, an aluminum hydroxide gel suspension. We harvested the quadriceps muscle and sera 2 weeks after the second immunization. Results are representative of two experiments. (B) Histology of the injected quadriceps muscle of each group. We visualized the inoculum remaining in the muscle by aluminum-specific lumogallion staining, and inflammatory cells by hematoxylin and eosin (H&E) staining. (C and D) Comparison of the levels of inflammation and remaining inoculum among groups. Each closed circle represents an individual mouse, and the bars indicate the mean values for each group. Lumogallion staining was negative in the PBS group; aluminum was not detected (N.D). ∗*p* < 0.05. We semi-quantified the levels of inflammation and remaining inoculum in the muscle. The inflammatory or lumogallion-positive areas were divided by the whole muscle areas to obtain the percentage of inflammatory or aluminum-containing areas. (E) Changes in body weight of immunized mice. The *y* axis represents the body weight (g) of mice, and the *x* axis represents the days after the first immunization. (F and G) Levels of S-RBD-specific (F) and JE-prME-specific (G) IgG in sera. Sera were diluted 2-fold from a 100-fold dilution, and IgG levels against each antigen were detected by enzyme-linked immunosorbent assays (ELISA). Thin lines represent the IgG levels of individual mice; open circles and bold lines represent the mean IgG values of each group. Error bars represent standard deviation (*n* = 5). (H) Serum neutralizing activities against SARS-CoV-2 Wuhan (D614G) strain pseudovirus. Serially 2-fold diluted sera were reacted with the pseudovirus, followed by infection of VeroE6-TMPRSS2 cells and measurement of NanoLuc luciferase activity in cell lysates 48 h later. (I) Serum neutralizing activities against JEV. Serially 2-fold diluted antisera were reacted with the JEV-NS1-HiBiT reporter virus, followed by infection of Vero cells and measurement of HiBiT activity in culture supernatants 72 h later.

Next, we evaluated whether JEprME-S-RBD nanoparticles could be used as vaccines. Four groups of BALB/c mice (*n* = 4–6 per group) were intramuscularly injected with vehicle (roup 1), 10 μg of soluble S-RBD (group 2), 1 μg of JEprME-S-RBD nanoparticles containing 1 μg of S-RBD (group 3), or a mixture containing corresponding amount of S-RBD and JEprME nanoparticles without bound ligand (mixture, group 4), each with an alum adjuvant, at intervals of 2–3 weeks, twice. Two weeks after the second injection, the muscle and sera were harvested from the injection sites (Figure 4A). Mice injected with PBS were used as negative controls (*n* = 3); the inoculum was an aluminum hydroxide suspension (alum) in all groups, except the mice that received PBS. We detected inflammatory cells and the remaining inoculum in the muscle using hematoxylin and eosin and aluminum-specific lumogallion staining, respectively. We observed similar levels of inflammation and remaining inoculum at the injection sites in all groups, except for the PBS-injected group (Figure 4B, left two). We often detected lumogallion-positive aluminum intracellularly, indicating that the inoculum was internalized by inflammatory cells at the injection sites (Figure 4B, right). We found no significant differences in the areas of inflammation or the areas, intensity, or load of lumogallion-positive aluminum among groups 1–4 (Figures 4C and 4D). We also found no significant differences in body weight between the groups during the observation period (Figure 4E). We compared serum antigen-specific immunoglobulin (Ig) G levels among the groups using enzyme-linked immunosorbent assay (ELISA) and found that mice immunized with JEprME-S-RBD particles (group 3) had significantly higher levels of S-RBD-specific antibodies than those immunized with mixture containing the same amounts of soluble S-RBD and JEprME antigens (group 4) (Figure 4F). Although group 3 received only 1 μg of the S-RBD protein contained in the JEprME-S-RBD particles, the high levels of S-RBD-specific antibodies were induced comparable to mice received 10 μg of soluble S-RBD (group 2). Group 3 also showed high levels of specific antibodies against the JEV carrier particles (Figure 4G).

We also assessed serum-neutralizing ability against SARS-CoV-2 using a pseudotyped virus of Wuhan strain. Only one of five sera from mice immunized with 10 μg of the soluble S-RBD protein (group 2) had a substantial neutralizing ability against SARS-CoV-2. In contrast, all sera from mice immunized with JEprME-S-RBD particles (group 3) exhibited higher neutralizing ability (Figure 4H). These results suggest that, by presenting the S-RBD antigen on the surface of the JEprME particles, a high level of immunogenicity can be achieved, which cannot be attained by immunization with the S-RBD protein alone. Using a reporter JEV with a HiBiT tag inserted at position 349Q of NS1,^35^ we also determined its serum-neutralizing ability against JEV. Sera from mice immunized with JEprME-S-RBD particles (group 3) and the mixture containing the same amount of JEprME antigen (group 4) showed high levels of neutralizing ability against JEV (Figure 4I). These results confirm that the immunogenicity of JEV was maintained even after binding to the S-RBD antigen.

### T cell responses to JEV and SARS-CoV-2 RBD

**Figure 5:**
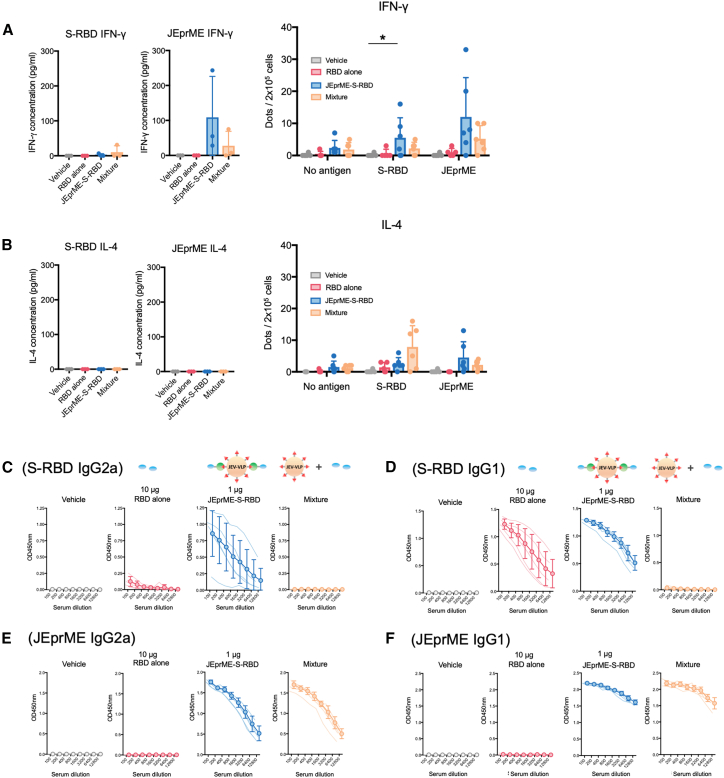
T cell responses to JEV and SARS-CoV-2 antigens (A) Production of interferon (IFN)-γ from lymph node cells harvested from immunized mice. Upon restimulation of lymph node cells with the S-RBD (left) or JEprME (middle), we quantified the amount of IFN-γ in the supernatants by ELISA. We also quantified the spot number of IFN-γ-producing cells by enzyme-linked immunosorbent spot (ELISpot) assays (right). ∗*p* < 0.05. (B) Production of interleukin (IL)-4 from lymph node cells harvested from immunized mice. IL-4 ELISA (left and middle) and ELISpot assay (right). (C and D) Titrations of anti-SARS-CoV-2 S-RBD IgG2a (C) and IgG1 antibodies (D). (E and F) Titrations of IgG2a (E) and IgG1 (F) antibodies against JE-prME. Sera were diluted 2-fold from a 100-fold dilution, and IgG isotype levels against each antigen were titrated by ELISA. Thin lines represent IgG isotype levels of individual mice; open circles and bold lines represent the mean IgG isotype levels of each group. Error bars represent standard deviation (*n* = 5).

To investigate whether JEprME-S-RBD immunization could induce Th-cell immune responses, we isolated lymph node cells from immunized mice and conducted cytokine ELISA and enzyme-linked immunosorbent spot (ELISpot) assays (Figures 5A, 5B, and S2). Following stimulation of lymph node cells with the S-RBD or JEprME antigens, we quantified the cells producing interferon (IFN)-γ and interleukin (IL)-4. We found that JEprME-S-RBD-immunized mice (group 3) had a larger number of IFN-γ-producing cells in response to both antigens compared with mice immunized with a 10-fold amount of soluble S-RBD (group 2) or the mixture (group 4) (Figures 5A and S2). Thus, Th1-cell responses were induced when JEV nanoparticles were used as carriers. In contrast, in the IL-4 ELISA and ELISpot assays following stimulation with the S-RBD antigen, administration of the mixture (group 4) induced a higher number of IL-4-producing cells in the ELISpot assays than in the JEprME-S-RBD particles (group 3), although no IL-4 was detected in ELISA in any group (Figure 5B). Thus, humoral immunity-associated Th2 responses appeared to be induced more efficiently in mice injected with a mixture of antigens.

We also examined serum IgG2a and IgG1 antibody responses to JEV and SARS-CoV-2 using ELISA (Figures 5C–5F), as Th1 and Th2 cytokine responses contribute to IgG2a and IgG1 production, respectively. We found that the sera from mice immunized with a 10-fold amount of soluble S-RBD protein (group 2) contained high levels of IgG1 against S-RBD, with minimal IgG2a levels (Figures 5A and 5B). In contrast, sera from mice immunized with JEprME-S-RBD particles (group 3) had higher titers of both IgG2a and IgG1 antibodies against S-RBD (Figures 5C and 5D). We also found that mice immunized with SpyCatcher-S-RBD exhibited substantial IgG1 production with minimal IgG2a in serum samples with no IFN-γ-producing cells in the lymph nodes. (Figure S3). Thus, the immune responses induced by immunization with soluble S-RBD protein alone were mostly Th2 responses; those induced by JEprME-S-RBD particles were both Th1 and Th2 responses.

On the other hand, we determined IgG isotype responses against the nanoparticle carrier, JEV particles. We found high levels of both IgG1 and IgG2a responses against JEprME in mice immunized with JEprME-S-RBD particles (group 3) and the mixture (group 4) but not in mice immunized with soluble S-RBD particles (group 2) (Figures 5E and 5F).

### Serum neutralizing abilities against various SARS-CoV-2 mutants

**Figure 6:**
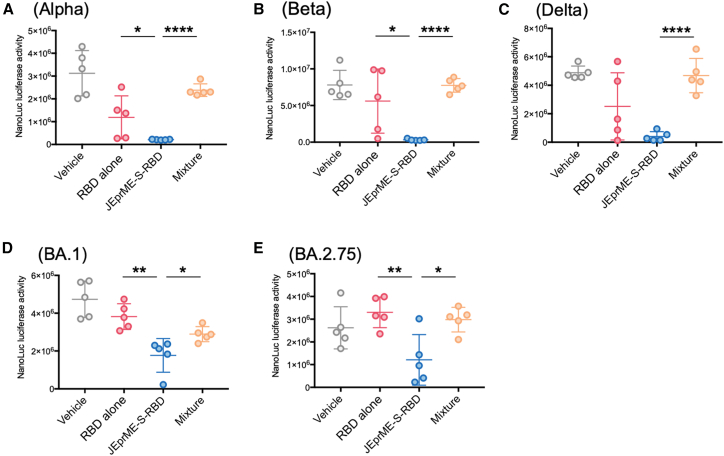
Serum neutralization activities against five SARS-CoV-2 variants Serum neutralization assays were conducted against pseudoviruses of the Alpha variant (A), Beta variant (B), Delta variant (C), Omicron BA.1 variant (D), and Omicron BA.2.75 variant (E). After reacting each pseudovirus with sera from each group, VeroE6-TMPRSS2 cells were infected, and luciferase activities were measured in cell lysates 48 h post-infection. Sera were diluted 2-fold from the original concentration. Each circle represents one mouse (*n* = 5), and bars indicate the mean values for each group. ∗*p* < 0.05, ∗∗*p* < 0.01, ∗∗∗*p* < 0.001, and ∗∗∗∗, *p* < 0.0001.

In this study, we constructed JEprME-S-RBD using the S-RBD of the Wuhan strain of SARS-CoV-2 and tested whether JEprME-S-RBD immunization could induce serum neutralizing antibodies against other SARS-CoV-2 variants (Figure 6). Pseudotyped viruses carrying the S proteins of SARS-CoV-2 Alpha (B.1.1.7), Beta (B.1.351), Delta (B.1.617.2), Omicron BA.1 (B.1.1.529), and Omicron BA.2.75 (BA.2.75 22D) variants were incubated with murine sera from groups 1 to 4 and then infected with VeroE6-TMPRSS2 cells. Among the four groups, the serum samples from mice immunized with JEprME-S-RBD particles (group 3) exhibited the strongest neutralizing abilities, not only against the Wuhan strain (Figure 4H), but also against the Alpha, Beta, and Delta variants (Figures 6A–6C). Significantly higher neutralizing abilities were also observed against Omicron BA.1 and Omicron BA.2.75 variants in murine sera from the JEprME-S-RBD particle immunization group than in those from the other three groups (Figures 6D and 6E). Thus, the conjugation of S-RBD to JEV nanoparticles seemed to enhance antigenicity, leading to the induction of neutralizing antibody responses against various mutant strains.

### Development of JEprME nanoparticles conjugating B19V RBD

**Figure 7:**
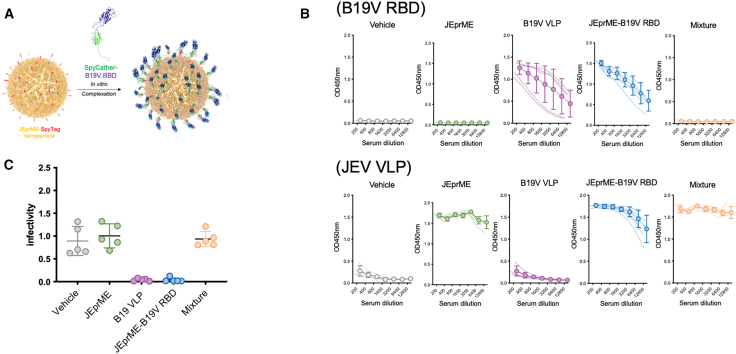
Immunization of B19V RBD conjugated JEV nanoparticles efficiently induced anti-B19V IgG and neutralizing antibodies (A) Schematic diagram of B19V VP1-RBD binding to JE-prME nanoparticles via SpyTag-SpyCatcher interaction. JE-prME, orange; SpyTag, red; B19V RBD, blue; and SpyCatcher: green. (B) Levels of B19V-specific (upper panels) and JE-prME-specific (lower panels) IgG in the sera. BALB/c mice (*n* = 5) were intramuscularly injected with PBS (group 1), 6.5 μg of JEprME nanoparticle (group 2), 5 μg of B19V VLPs (group 3), 10 μg of JEprME-B19V RBD nanoparticles containing S-RBD (group 4), corresponding amount of non-binding mixture (JEprME-SpyTag and soluble B19V RBD) (group 5) with alum adjuvant at 2-week intervals. Sera were collected 2 weeks after the second immunization. Sera were diluted 2-fold from a 200-fold dilution, and antibody levels against each antigen were detected by ELISA. Thin lines represent the antibody levels of individual mice; open circles and bold lines represent the mean values. Error bars represent standard deviation (*n* = 5). (C) Evaluation of serum neutralizing antibodies against B19V. Murine sera were mixed with B19V-positive human plasma from blood donors and incubated at 4°C for 1 h. The antibody-virus mixtures were then introduced to UT7/Epo-S1 cells and cultured for 72 h. After permeabilization, the cells were stained with an anti-VP antibody. Neutralization assays were carried out using sera from each group. The *y* axis in the graph shows the relative number of VP-positive cells, with individual circles representing sera from each group. Error bars reflect the standard deviation of the dataset relative to the mean. Results are representative of two experiments.

To demonstrate the potential of JEV nanoparticles as carriers for multivalent vaccines targeting various infectious diseases, the B19V RBD was coupled with JEV nanoparticles. The SpyCatcher-fused B19V VP1u 5–80 aa fragment was purified and conjugated to JEprME-SpyTag. Following conjugation, the antigen-coupled nanoparticles were purified by sucrose density gradient centrifugation to remove free unconjugated ligands (Figure S4). BALB/c mice were intramuscularly injected with vehicle (group 1), 6.5 μg of JEprME nanoparticles (group 2), 5 μg of B19V VLPs (group 3), 10 μg of JEprME-B19V RBD nanoparticles containing 3.5 μg of B19V RBD (group 4), or a mixture containing corresponding amount of B19V RBD and JEprME nanoparticles without bound ligand (mixture, group 5), each in an alum adjuvant, at two-week intervals, twice. Two weeks after the second administration, serum antigen-specific IgG levels were measured using ELISA. Mice immunized with JEprME-B19V RBD nanoparticles (group 4) and B19V VLPs (Group 3) had significantly higher levels of specific antibodies against the B19V RBD than mice immunized with the mixture (group 5) (Figure 7B). We also found high levels of specific antibodies against carrier JEV particles (Figure 7B).

We assessed the neutralizing ability of sera against B19V infection in UT7/Epo-S1 cells. We found high serum-neutralizing abilities against B19V in mice immunized with B19V VLPs (group 3) or JEprME-B19V RBD nanoparticles (group 4), but not in the unconjugated mixture (group 5) (Figure 7C). Thus, presenting the B19V RBD antigen on the surface of JEprME nanoparticles significantly enhanced immunogenicity. Here, we demonstrate that JEV nanoparticles could serve as effective carriers for multivalent vaccines targeting various viral infectious diseases, in addition to COVID-19.

## Discussion

Protein nanoparticle vaccines have been experimentally demonstrated to be novel modalities for inducing antimicrobial immune responses.^36^ Since protein nanoparticle vaccines comprise microbial antigens and carrier proteins such as ferritin and lumazine synthase, these vaccines have been shown to induce immune responses not only against microbes but also against carrier proteins.^12^ Immune responses to carrier proteins reduce vaccine efficacy.^1^ To address this issue, we designed flavivirus-VLPs carrying microbial antigens as novel nanoparticle vaccines. We hypothesized that the induction of anti-JEV responses could rather enhance, not lessen, the immunogenicity against the targeted microbial antigens, since repeated anti-microbial immunizations have been known to induce more active and lasting immune responses over time.^37^

JEV belongs to the genus *Flavivirus* and is transmitted by mosquitoes, particularly *Culex tritaeniorhynchus* species, causing severe viral encephalitis known as Japanese encephalitis.^38^^,^^39^ Its natural host is pigs, and human infections occur when JEV-carrying mosquitoes bite humans. The incubation period of JEV is 6–16 days, and encephalitis occurs after the onset of symptoms, such as fever and headache, with a high mortality rate of 20%–40%. Even if recovery occurs, 45%–70% of cases result in severe sequelae due to neurological damage. In some countries, vaccination with three doses for children aged 3–7 years and one dose for children aged 9–13 years, for a total of four doses of inactivated vaccine, has been highly successful in preventing JEV infection.^40^ These findings confirm the safety of JEV nanoparticle-based vaccines. In addition to JEV, other flaviviruses, including YFV, ZIKV, WNV, and TBEV, could also serve as potential candidates for carrier nanoparticles, although DENV may be inappropriate because of the risk of antibody-dependent enhancement of viral infection.^41^

Previously, Saga et al. identified several insertion sites for foreign epitopes on the surface of JEV particles, particularly on the JEV E protein. The authors incorporated hepatitis C virus (HCV)-neutralizing epitopes into JEV-VLPs.^23^ Although mice immunized with HCV/JEV VLPs mounted neutralizing antibodies against JEV and HCV, the anti-HCV antibody titers measured by ELISA were lower than those in mice immunized with HCV peptide alone. The JEV E ectodomain contains 11 loops.^33^ In this study and our previous study, we introduced a highly sensitive detection tag system, the HiBiT tag, which enabled us to comprehensively identify peptide insertion sites in the JEV E protein.^32^ In addition to the loop starting from Ser 275 residue reported previously,^23^ we found that the loop near Pro 228 residue allowed for peptide insertion (Figure 2C). Furthermore, attaching a GS linker efficiently increased particle production when the peptide was inserted into the Ser 275 loop (JEprME-SpyTag) (Figure 2C). These modifications allowed us to successfully produce large quantities of nanoparticles, using a mammalian cell expression system, which has the potential for industrial applications.

In this study, we used JEprME-S-RBD particles containing the ligand antigen and JEV particles at an approximate ratio of 1:32 (Figure 3D). Although this represents a relatively low coupling efficiency, the nanoparticles effectively induced the production of neutralizing antibodies against SARS-CoV-2 and JEV. This may be attributed to the limited number of conjugated ligands, which likely preserved the exposure of key epitopes on the JEV nanoparticles. The coupling efficiency is influenced by factors such as reaction time and protein concentration in the reaction mixture. Further optimization of these conditions may be necessary to improve the coupling efficiency and enhance the immunogenicity of the resulting nanoparticles.

The substantial S-RBD antibody response observed after JEprME-S-RBD immunizations (Figure 4F) can be explained by the induction of antigen-specific Th cells. By ELISpot assays, we found that the mice immunized with the JEprME-S-RBD nanoparticles had the substantial numbers of both IFN-γ- and IL-4-producing T cells specific for either S-RBD or JEprME, compared with mice injected with vehicle or S-RBD (Figures 5A and 5B). These results were consistent with the findings that only JEprME-S-RBD immunization resulted in significant production of both IgG1 and IgG2a isotypes (each isotype response reflecting Th2 and Th1 cytokine responses, respectively) against S-RBD, although S-RBD immunization induced IgG1, but not IgG2a isotype responses against S-RBD (Figures 5C–5F). On the other hand, the mice immunized with the mixture of JEV-VLP and S-RBD also had IFN-γ- and IL-4-producing T cells specific for either S-RBD or JEprME, although the mice mounted IgG1 and IgG2a isotype responses only to JEprME, but not S-RBD.

In mice immunized with JEprME-S-RBD particles, we observed the induction of Th1-cytokine-producing cells (Figure 5A). Although protein antigens are thought to induce Th2-biased immune responses, other nanoparticles, including silica, often do not efficiently induce cellular immune responses.^42^ Thus, unlike other protein antigens or nanoparticles, JEprME-S-RBD nanoparticles seemed to efficiently induce Th2 and Th1 responses via unknown mechanism(s). Cellular immune responses, characterized by Th1 responses, have been shown to play a central role in defense against viral infections and contribute to long-term immune memory.^43^ Thus, our novel protein-based JEprME-S-RBD nanoparticles are expected to function efficiently as vaccines inducing antiviral immune responses.

JEprME-S-RBD immunization induced linked recognition as per our working hypothesis (Figure 1), supported by a significant anti-S-RBD antibody response in the JEprME-S-RBD-immunized group, but not in the JEV-VLP/S-RBD-immunized group. In the current experiment, JEprME-S-RBD immunized mice had six S-RBD-specific and 10 JEprME-specific IFN-γ-producing cells in total 2 × 10^5^ cells; the total number of IFN-γ-producing cells was 16, all of which could help both S-RBD-specific and JEprME-specific antibody productions (Figures 5A and S3). On the other hand, mice immunized with the JEV-VLP/S-RBD mixture had three S-RBD-specific and five JEprME-specific IFN-γ-producing cells, each of which were the T cell help in the production of S-RBD-specific and JEprME-specific antibodies, respectively (Figures 5A and S3). The higher number of IFN-γ-producing Th cells led to an optimal antibody response in JEprME-S-RBD immunizations. These results indicate that JEprME nanoparticles may play a role in enhancing the antibody response to S-RBD. Furthermore, it is possible that JEprME nanoparticles not only served as an antigen but also played an adjuvant-like role. These findings suggested that linked recognition may be a promising strategy in vaccine design. Although this study examined the impact of the number of IFN-γ-producing cells on antibody responses, other cytokines may also play important roles. Further analysis of these factors may help elucidate a more comprehensive immune response mechanism.

In this study, immunization with JEprME-S-RBD induced neutralizing antibodies against the Wuhan strain and various SARS-CoV-2 variants (Figures 6A–6E). This could be attributed to the highly potent immunogenicity achieved through the particle formation of the S-RBD antigen, which was not achievable by conventional immunization with soluble S-RBD protein. The induction of neutralizing antibodies against various mutant strains indicated the potential of our nanoparticle vaccine as a universal vaccine. Linked recognition may also contribute to the potent immunogenicity of the nanoparticle vaccines. Linked recognition was discovered through studies on the production of antibodies against haptens; when haptens were coupled to a carrier protein, they became immunogenic, a phenomenon known as the hapten-carrier effect.^44^ Several vaccine designs exploit linked recognition. For example, the *Haemophilus influenzae* type b (Hib) vaccine was a conjugate of the Hib polysaccharide and tetanus toxoid protein; toxoid-specific T cells produced cytokines, helping to produce Hib-specific antibodies.^45^ Because B cells, whose B cell receptors bind to specific antigens, can be 10,000 times more efficient at displaying epitopes of those antigens on their major histocompatibility complex (MHC) class II molecules,^46^^,^^47^ S-RBD epitopes incorporating JEprME-S-RBD nanoparticle vaccines could likely be presented more efficiently on MHC class II molecules.

Our novel JEV nanoparticle vaccine design is applicable as a carrier for multivalent vaccines that can target various infectious diseases by incorporating neutralizing epitopes of pathogens, including B19V (Figure 7). Previously, we demonstrated that the conjugation of B19V RBD to ferritin or I3-01, non-viral self-assembling nanoparticles, significantly enhanced the immunogenicity of this antigen.^29^ Our current results, which utilized coupling to JEprME nanoparticles, were consistent with previous findings. Since B19V spread is commonly observed among children, conjugating the B19V RBD to the JEV vaccine and administering the conjugated vaccine to children in the high-risk group would be an efficient approach for preventing oB19V infection. Thus, B19V RBD-coupled JEprME nanoparticles may serve as strong candidates for bivalent vaccines to prevent both JEV and B19V infections.

In the future, it will be possible to create cells that stably express SpyCatcher-RBD, allowing the infection of these cells with Tag-inserted recombinant JEV to produce RBD-JEV nanoparticles through autonomous replication, which would offer the advantage of an easy transition to large-scale cultivation. Moreover, by leveraging the manufacturing process of previously produced inactivated JEV vaccines, vaccine production can be simplified and accelerated, facilitating straightforward and time-efficient vaccine manufacturing.

### Conclusion

In this study, we developed a JEV nanoparticle vaccine incorporating the SARS-CoV-2 S-RBD antigen on the surface of JEV-VLPs, anticipating the linked recognition of the two viral antigens, thereby Th cells specific to each virus would help to enhance antibody responses to both viruses. We compared anti-JEV and S-RBD antibody production and Th cytokine production between (1) JEprME-S-RBD nanoparticle vaccines, (2) the S-RBD vaccine alone, and (3) a mixture of JEV-VLP and S-RBD vaccines. We found that only JEprME-S-RBD nanoparticle immunization induced robust neutralizing antibody responses against SARS-CoV-2. We also found that JEprME-S-RBD nanoparticle immunizations induced JEV- and SARS-CoV-2-specific IFN-γ- and IL-4-producing Th cells. Finally, we demonstrated that the coupled antigen could be replaced with epitopes from different pathogens, such as B19V VP1-RBD. Therefore, our novel JEprME-coupled nanoparticle vaccine represents a promising bivalent vaccine capable of simultaneously preventing JEV and other pathogens linked to these nanoparticles.

### Limitations of the study

Our study has several limitations. First, we did not examine whether our novel vaccines could protect against viral infection *in vivo*, such as by using human ACE2 transgenic mice. When such *in vivo* protection is observed, it is important to determine whether the protection can be mediated by serum-neutralizing antibodies or other effectors, such as anti-viral cytotoxic T cell responses. In translational research, a long-term observation experiment after the final vaccination may be crucial to determine how long our novel vaccination remains protective by evaluating anti-viral IgG memory responses as well as virus-specific T cell proliferation.

## Resource availability

### Lead contact

Requests for further information and resources should be directed to and will be fulfilled by the lead contact, Eiji Morita (moritae@hirosaki-u.ac.jp).

### Materials availability

All unique/stable reagents generated in this study are available from the lead contact with a completed materials transfer agreement.

### Data and code availability

Any additional information required to reanalyze the data reported in this paper is available from the lead contact upon request.

## Acknowledgments

We thank Ryusei Tsushima for helping with the electron microscopic observations. This research was supported by 10.13039/501100001691JSPS 10.13039/501100001691KAKENHI (grant numbers 23790503, 26460555, 16H01188, 17H06413, 20K21874, 22K18378, 22H02873, 22H00553, and 24K10163); JST 10.13039/501100003382CREST, Japan (grant number JPMJCR17H4); 10.13039/100009619AMED, Japan (grant number 20339008, 20333747, 19fk0108168h0001, 20he0622012h0001, and 22fk0108527s0101); and 10.13039/501100019670The Research Foundation for Microbial Diseases of Osaka University (BIKEN).

## Author contributions

Conceptualization, E.M.; data curation, K. Ii; formal analyses, K. Ii, C.T.N., Y.H., H.S., and K. Ishida; funding acquisition, E.M.; investigations, K. Ii, C.T.N., Y.H., H.S., K. Ishida, and A.Y.; methodology, K. Ii, C.T.N., H.S., and K. Ishida; project administration, N.T., I.T., H.E., and E.M.; resources, M.A., supervision, E.M. and F.S.; validation, T.N., K.N., and R.I.; visualization, K. Ii, C.T.N.; writing – original draft, E.M.; writing – review and editing, F.S., I.T., and E.M.

## Declaration of interests

H.S., T.N., A.Y., and H.E. are employees of BIKEN.

## STAR★Methods

### Key resources table

**Table:** 

REAGENT or RESOURCE	SOURCE	IDENTIFIER
**Recombinant DNA**		

Plasmid: pCAG-JEprME(RS1228) CtermGS-HiBiT	Ishida et al.^32^	N/A
Plasmid: pCAG-JEprME[N47-spytag-V56] CtermGS-HiBiT	-	N/A
Plasmid: pCAG-JEprME[N47-spytag-V56] CtermGS-HiBiT	-	N/A
Plasmid: pCAG-JEprME[M45-spyTag-R56] CtermGS-HiBiT	-	N/A
Plasmid: pCAG-JEprME[L41-spytag-E55] CtermGS-HiBiT	-	N/A
Plasmid: pCAG-JEprME[P75-spytag-S88] CtermGS-HiBiT	-	N/A
Plasmid: pCAG-JEprME[G127-spytag-Y137]CtermGS-HiBiT	-	N/A
Plasmid: pCAG-JEprME[P171-spytag-P182] CtermGS-HiBiT	-	N/A
Plasmid: CAG-JEprME[P75-spytag-GS-S88] CtermGS-HiBiT	-	N/A
Plasmid: pCAG-JEprME[H152-spytag-A161] CtermGS-HiBiT	-	N/A
Plasmid: pCAG-JEprME[ P228-spytag-S229]CtermGS-HiBiT	-	N/A
Plasmid: pCAG-JEprME[ P228-GS-spytag-GS-S229]CtermGS-HiBiT	-	N/A
Plasmid: pCAG-JEprME[P227-C-spytag-C-S230]CtermGS-HiBiT	-	N/A
Plasmid: pCAG-JEprME[P227-GS-C-spytag-GS-C-S230] CtermGS-HiBiT	-	N/A
Plasmid: pCAG-JEprME[P228-spytag-M240]CtermGS-HiBiT	-	N/A
Plasmid: pCAG-JEprME[S230-GS-C-spytag-GS-C-A233]CtermGS-HiBiT	-	N/A
Plasmid: CAG-JEprME[S230-C-spytag-C-W233] CtermGS-HiBiT	-	N/A
Plasmid: CAG-JEprME[T231-spytag-A232]CtermGS-HiBiT	-	N/A
Plasmid: pCAG-JEprME[S230-C-spytag-C-W233]CtermGS-HiBiT	-	N/A
Plasmid: pCAG-JEprME[S230-GS-C-spytag-GS-C-W233]CtermGS-HiBiT	-	N/A
Plasmid: pCAG-JEprME[T231-GS-spytag-GS-A232]CtermGS-HiBiT	-	N/A
Plasmid: pCAG-JEprME [T231-spytag-L239]CtermGS-HiBiT	-	N/A
Plasmid: pCAG-JEprME[L266-spytag-S276]CtermGS-HiBiT	-	N/A
Plasmid: pCAG-JEprME[L266-GS-spytag-GS-S276]CtermGS-HiBiT	-	N/A
Plasmid: pCAG-JEprME[S275-spytag-S276]CtermGS-HiBiT	-	N/A
Plasmid: pCAG-JEprME[Y274-C-spytag-C-S277]CtermGS-HiBiT	-	N/A
Plasmid: pCAG-JEprME[S275-GS-spytag-GS-S276]CtermGS-HiBiT	-	N/A
Plasmid: pCAG-JEprME[T300-spytag-K307]CtermGS-HiBiT	-	N/A
Plasmid: pCAG-JEprME[T231-spytag-L239]CtermGS-HiBiT	-	N/A
Plasmid: pCAG-JEprME[S364-spytag-S364]CtermGS-HiBiT	-	N/A
Plasmid: pCAG-JEprME[S275-GS-spytag-GS-S276]	-	N/A
Plasmid: pCAG-JEprME-D67N-[S275-GS-spytag-GS-S276]	-	N/A
Plasmid: pcDNA3.1-MycHis(-)A	-	N/A
Plasmid: pcDNA3.1-FLAG--HiBiT	-	N/A
Plasmid: pcDNA3.1-SARS2-Spike	-	N/A
Plasmid: pcDNA3.1-SARS2-Spike_D614G_del20	-	N/A
Plasmid: pcDNA3.1-SARS2-Spike-Alpha del20-FLAG--HiBiT	-	N/A
Plasmid: pcDNA3.1-SARS2-Spike-Beta del20-FLAG--HiBiT	-	N/A
Plasmid: pcDNA3.1-SARS2-Spike-Delta del20-FLAG--HiBiT	-	N/A
Plasmid: pcDNA3.1-SARS2-Spike-Omicron BA.1	-	N/A
Plasmid: pcDNA3.1-SARS2-Spike-Omicron BA.2.75 del20-FLAG-HiBiT	-	N/A
Plasmid: pcDNA3.1_Kz-IL2SP-His(6×)-GS-CoV2_RBD_(hu)	-	N/A
Plasmid: pcDNA3.1_Kz-IL2SP-His(6×)-GS-SpyCatcher-GS-CoV2_RBD_(hu)	-	N/A
Plasmid: pQC-Nluc-IP	-	N/A
Plasmid: pUC19.JEV_NS1-349-HiBiT4GS	-	N/A
Plasmid: gag-pol	-	N/A
Plasmid: hACE2	Li et al.	Addgene Plasmid: #1786
Plasmid: TMPRSS2	Edie et al.	Addgene Plasmid: #53887

**Antibodies**		

Rabbit monoclonal anti-SARS-CoV-2 RBD [HL257]	GeneTex	Cat# GTX635692; RRID: AB_2888564
Rabbit monoclonal anti-Japanese encephalitis virus Envelope	GeneTex	Cat# GTX125867; RRID: AB_11172365
Peroxidase AffiniPure Goat Polyclonal anti- Rabbit IgG (H+L)	Jackson ImmunoResearch	Cat# 111-035-003; RRID: AB_2313567
Peroxidase AffiniPure Goat Polyclonal anti- Mouse IgG (H+L)	Jackson ImmunoResearch	Cat# 115-035-003; RRID: AB_10015289
HRP conjugated Goat Polyclonal anti- Mouse IgG, Fcγ Subclass 1 Specific	Proteintech	Cat# SA00012-1; RRID: AB_2890964
HRP conjugated Goat Polyclonal anti- Mouse IgG, Fcγ Subclass 2a Specific	Proteintech	Cat# SA00012-2; RRID: AB_2890965

### Experimental model and study participant details

#### Cells

HEK293T cells, Vero cells, and VeroE6-TMPRSS2 cells^48^ were cultured in Dulbecco's Modified Eagle's Medium (DMEM) high glucose (Nacalai Tesque, Kyoto, Japan) containing 10% fetal bovine serum (FBS, Thermo Fisher Scientific Inc., Waltham, MA), 100 units/mL penicillin (Nacalai Tesque), and 100 μg/mL streptomycin (Nacalai Tesque) at 37°C with 5% CO_2_. Expi293F cells were maintained in a shaking culture at 37°C with 8% CO_2_ using Expi293 Expression Medium (Gibco). UT7/Epo-S1 cells were cultured in RPMI 1640 medium with 10% FBS, 100 μg/mL penicillin, and 100 units/mL streptomycin. Erythropoietin (Thermo Fisher Scientific Inc., Waltham, MA), a growth factor, was added to UT7/Epo-S1 cell culture medium at a concentration of 2 units/ml. All cell lines have been verified free from mycoplasma contamination.

#### Animals

We purchased 6-7 to week-old female BALB/c mice from CLEA Japan, Inc. (Tokyo, Japan) and maintained them under specific pathogen-free conditions in the animal care facility at the Kindai University Faculty of Medicine (Osaka, Japan) or Research Institute for Microbial Diseases, Osaka University (Osaka, Japan). The experimental procedures were approved by the Institutional Animal Care and Use Committee of the Kindai University Faculty of Medicine (protocol number: KAME-2022-001, 2022) and Osaka University (protocol number: R02-10-0. 2020), and were conducted according to the criteria outlined by the National Institutes of Health (NIH).

### Method details

#### Construction of plasmid DNA and transfection into cultured cells

All plasmid DNA used in this study were constructed using either ligation or Gibson assembly of polymerase chain reaction (PCR) products. Information regarding all the plasmids used in the experiments is provided in the key resources table. Plasmid transfection into HEK293T cells was performed using Polyethylenimine Max (PEI Max; 40,000 Da; Polysciences Inc., Warrington, PA, USA) or the Lipofectamine 3000 Transfection Kit (Thermo Fisher Scientific Inc.). Plasmid transfection into Expi293F cells was performed using PEIMax or an ExpiFectamine 293T Transfection Kit (Thermo Fisher Scientific Inc.). After adding the reagent and plasmid DNA mixture to the cultured cells, the medium was replaced after 6 h, and the culture supernatants and cells were collected at the desired time points.

#### Measurement of HiBiT dependent nanoLuc luciferase activity

HiBiT dependent nanoLuc luciferase activity (HiBiT activity) was detected using the Nano-Glo® HiBiT Lytic Detection System (Promega Corporation, Madison, WI) and Varioskan LUX Multimode Microplate Reader (Thermo Fisher Scientific Inc.) according to the manufacturer's protocol. The cells were lysed in lysis buffer (150 mM NaCl, 20 mM Tris (pH 7.5), and 1% Triton X-100) for 15 min. HiBiT Lytic buffer was prepared by mixing the LgBiT Protein and HiBiT Lytic Substrate at a 100:1 ratio to create the LgBiT solution. For measurement, 5 μL of the LgBiT solution was mixed with 5 μL of the sample in a 384-well White plate (Greiner Bio-One Co. Ltd., Tokyo, Japan) and allowed to react.

#### Preparation and purification of JEprME nanoparticles

JEprME (E-D67N)^32^ or JEprME-SpyTag (E-D67N), derived from Nakayama strain,^23^ was transiently expressed in Expi293F cells by plasmid transfection using PEI Max or ExpiFectamine transfection reagents. The culture supernatant was collected at 72 h post-transfection. After collecting, the supernatant was centrifuged twice at 1,000*g* for 10 min at 4°C, and then concentrated by ultrafiltration [Amicon stirred cell, 200 mL (Merck Millipore, Burlington, MA), Ultracel 100 kDa Ultrafiltration Discs (Merck Millipore)]. The resulting concentrates were fractionated using 15–60% (w/v) sucrose density gradient ultracentrifugation, and the target proteins were fractionated using Coomassie brilliant blue (CBB) staining and western blotting. Subsequently, the fractions containing the JEprME nanoparticles were collected, dialyzed overnight using PBS to remove sucrose, and concentrated again using ultrafiltration.

The SpyCatcher-fused SARS-CoV-2 S-RBD contains a signal peptide derived from IL-2 and a cleavable His tag for immobilized metal affinity chromatography (IMAC) purification using Ni-NTA resin. Soluble S-RBD and SpyCatcher-RBD were transiently expressed in Expi293F cells by plasmid transfection using PEI-Max or ExpiFectamine transfection reagents. On the 8th day post-transfection, the supernatant was collected and filtered through a 0.22 μm filter [sartolab BT150 Filter System 12(48)/CS, size: 0.22 μm PES, 150 mL]. Subsequently, the supernatant was loaded onto a column packed with Ni-affinity beads (Ni-Sepharose 6 Fast Flow; GE Healthcare) equilibrated with Ni-equilibrating buffer [10 mM Tris-HCl (pH 8.0), 400 mM NaCl, 5 mM MgCl_2_, and 10% glycerol]. Washing buffer [10 mM Tris-HCl (pH 8.0), 400 mM NaCl, 5 mM MgCl_2_, 10% glycerol, and 20 mM imidazole] was used for column washing, followed by elution using elution buffer [10 mM Tris-HCl (pH 8.0), 400 mM NaCl, 5 mM MgCl_2_, 10% glycerol, and 1 M imidazole] at a flow rate of 0.5 mL/min. After separation by sodium dodecyl sulfate-polyacrylamide gel electrophoresis (SDS-PAGE), the samples were analyzed by CBB staining and western blotting. The eluted fractions containing the sample were collected and dialyzed overnight using ion-exchange A buffer [20 mM Tris-HCl (pH 8.0), 20 mM NaCl]. The dialyzed sample was passed through a syringe filter (0.22 μm, Azlon) and subjected to anion exchange column chromatography. The sample was loaded onto HiTrap Q Sepharose Fast Flow IEX columns (Cytiva) equilibrated with ion exchange buffer A and eluted by gradually increasing the salt concentration using ion exchange buffer B [20 mM Tris-HCl (pH 8.0), 1 M NaCl].

SpyCatcher-fused B19V VP1-RBD containing a cleavable His tag was prepared using a bacterial expression system. The *Escherichia coli* strain BL21-CodonPlus (DE3)-RIPL was transformed with the pCold-RBD-SpyCatcher plasmid and precultured overnight at 37°C in ZYM5052 medium. The culture was then transferred to the main medium and the absorbance was measured; when the optical density (OD) reached 0.3–0.6, the incubation temperature was lowered to 15°C to induce protein expression. After 24 h, the cells were harvested by centrifugation at 4,000*g* for 10 min. The collected cells were resuspended in buffer [10 mM Tris-HCl (pH 8.0), 400 mM NaCl, 5 mM MgCl_2_, 0.1 mM phenylmethylsulfonyl fluoride (PMSF), 1 mM 2-mercaptoethanol, and 10% glycerol] and disrupted by sonication. Following disruption, the lysate was centrifuged at 20,000*g* for 10 min to separate soluble and insoluble fractions. The B19V RBD-SpyCatcher in the soluble fraction was purified using Ni-affinity chromatography.

After detection by SDS-PAGE with CBB Blue staining and western blotting, the eluted fractions containing the sample were collected and dialyzed overnight in PBS. The sample was concentrated using ultrafiltration (Vivaspin 20, 10,000 MWCO, 29 mm diameter, Sartorius).

To conjugate ligands to the nanoparticles, purified JEprME-SpyTag nanoparticles and SpyCatcher-fused SARS-CoV-2 S-RBD or SpyCatcher-fused B19V VP1-RBD were mixed in a molar ratio1:1 for 8 h at 25°C. The mixtures were fractionated using 15–60% (w/v) sucrose density gradient ultracentrifugation. Subsequently, the fractions containing the JEprME nanoparticles were collected, dialyzed overnight using PBS to remove sucrose, and concentrated again using ultrafiltration.

#### Preparation and purification of B19V VLPs

B19V VLPs were prepared and purified as described previously.^29^ Briefly, the pCAG-optVP1 and pCAG-optVP2 vectors were transfected into HEK293T cells. After 24 h, the cells were harvested and lysed via three freeze–thaw cycles in PBS. After centrifugation at 20,000*g* for 10 min at 4°C, the supernatant fraction was subjected to ultracentrifugation on a 15%–30% (w/v) sucrose density gradient at 220,000*g* at 4°C for 3 h. After removing sucrose via dialysis, the VLP-positive peak fractions were further purified using Q-Sepharose (Cytiva, Marlborough, MA) column chromatography. After binding to Q-Sepharose using a binding buffer [50 mM Tris-HCl (pH 7.5), 0.1 M NaCl], the column was washed with 10 times the column volume of the binding buffer. Bound VLPs were eluted via linear gradient elution using an elution buffer [50 mM Tris-HCl (pH 7.5), 1.0 M NaCl]. After removing NaCl via dialysis, the VLPs were concentrated by ultrafiltration (pore size 0.22 μm; Merck Millipore). Dynamic light scattering measurements were performed using a Malvern Zetasizer Nano ZS particle size analyzer (Malvern Instruments Ltd., Worcestershire, UK).

#### Electron microscopy

The samples for electron microscopy were prepared using carbon film grids (Colloidal Film Attachment Mesh NISSHIN EM Co. Ltd., Tokyo, Japan: JB12A). The grids were placed face-up on a glass slide and hydrophilized by discharge for 40 seconds (s). The grids were then placed on a resin plate, and 5 μL of the sample was added and allowed to stand for 10 min. After removing the excess solution with filter paper, 5 μL of EM Stainer (NISSHIN EM) diluted four times with ultrapure water was added to the grid film for negative staining. After 5 min, the excess solution was removed, and the grids were allowed to dry on the resin plate until observation. Observations were conducted at 200 kV using a transmission electron microscope (JEM-2100; JEOL, Tokyo, Japan).

#### Immunization

For JEprME-S-RBD immunization experiments, we prepared the following antigens: the S-RBD protein (10 μg/mouse), JEprME-S-RBD nanoparticles (33 μg/mouse, consisting of S-RBD: 1 μg/mouse and JEprME-SpyTag: 32 μg/mouse), and a mixture (33 μg/mouse). For JEprME-B19V RBD *in vivo* experiments, we prepared the following antigens: the B19V VLPs (5 μg/mouse), JEprME (6.5 μg/mouse), JEprME-B19V RBD nanoparticles (10 μg/mouse, consisting of B19V RBD: 3.5 μg/mouse and JEprME-SpyTag: 6.5 μg/mouse), and a mixture of the B19V RBD protein (1.2 μg/mouse) and JEprME-SpyTag (6.5 μg/mouse) without conjugation. These antigens were mixed with PBS and 2% Alhydrogel adjuvant (alum, an aluminum hydroxide gel suspension, InvivoGen, San Diego, CA, USA). The final concentration of aluminum in the antigen solutions was 500 μg/mL (50 μg per mouse). We injected 8-week-old female BALB/c mice intramuscularly with 100 μL of each inoculum. The Vehicle group was administered alum solution alone as a control. Each group comprised five to six mice per experiment. We monitored body weight changes during the observation period.

#### Antibody ELISAs

We killed the immunized mice 2–3 weeks after the second immunization and collected blood from their hearts. We centrifuged the blood samples at 2775*g* at 4°C for 20 min, harvested and stored the sera at –80°C until examination. We coated a 96-well ELISA Flat Bottom Plate (IWAKI; 3801-096, AGC TECHNO GLASS CO., LTD., Tokyo, Japan) with 50 μL/well of the JEprME (1 μg/mL) or SARS-CoV-2 S-RBD (1 μg/mL) or B19V VLP (1 μg/mL) antigen and incubated the plate overnight at 4°C for immobilization. After discarding the antigen, the plate was washed three times with a washing buffer (0.05% Tween20 in PBS) followed by blocking with an assay diluent [1% bovine serum albumin (BSA) in PBS] for 2 h at 25°C. Then, we washed the plate three times with the washing buffer and added 50 μL of the serum samples to the wells. Serum samples were diluted with the assay diluent in two-fold serial dilutions, starting from a 100-fold dilution. After the 2-h incubation at 25°C, we washed the plate three times with the washing buffer, added a horseradish peroxidase (HRP)-conjugated anti-mouse IgG (H+L), IgG1, or IgG2a secondary antibody diluted with the assay diluent to the wells, and incubated the plate for 1 h at 25°C. Following five washes with the washing buffer, a BD OptEIA™ TMB substrate reagent (BD Biosciences, San Jose, CA) was added at 50 μL/well to detect the immunoreactive complexes for 5 min at 25°C. The color reaction was stopped by adding 0.5 M phosphate buffer. The absorbance was measured at 450 and 620 nm using a Varioskan LUX Multimode Microplate Reader (Thermo Fisher Scientific Inc., Waltham, MA, USA).

#### Serum neutralizing ability using JEV-NS1-HiBiT

Vero cells were seeded in 96-well plates at a density of 3 × 10^3^ cells/well. Sera were diluted two-fold and mixed with JEV-NS1-HiBiT [generated by circular polymerase extension reaction (CPER) method^32^^,^^35^] solution at a ratio of 1:1, and the antigen-antibody reaction was allowed to proceed for 2 h. The resulting mixture was used to infect Vero cells at a multiplicity of infection (MOI) of 0.001. After a 5-h incubation, the culture supernatant was removed, and the cells were washed and further cultured for 72 h. Subsequently, the culture supernatant was collected. The HiBiT activity in the culture supernatant was measured to quantify viral proliferation.

#### Serum neutralizing ability using SARS-CoV-2 pseudovirus

HEK293T cells were seeded in 6-cm dishes at a density of 4 × 10^5^ cells/well. The cells were transfected with vectors expressing nanoLuc luciferase-expressing retrovirus, retroviral gag-pol (Takara Bio USA, Inc. CA, USA), and plasmids encoding the SARS-CoV-2 S protein using PEI Max. After the 48-h cultivation, the supernatant was collected, debris was removed, and the resulting solution was used as a pseudovirus. Then, VeroE6-TMPRSS2 cells were seeded in 96-well plates at a density of 5 × 10^3^ cells/well. Sera were diluted two-fold, mixed with 5 μL of the pseudovirus solution and incubated for 2 h to allow antibody-mediated viral neutralization. The resulting mixture was used to infect VeroE6-TMPRSS2 cells, and the NanoLuc luciferase activity of the cells was measured after the 48-h cultivation.

#### Neutralizing antibody abilities for human parvovirus B19

Murine serum (5 μL) and B19V-positive human plasma (5 μL, containing 10^9^ B19V viral DNA copies per ml) were mixed and incubated at 25°C for 1 h. The B19V-positive plasma sample was obtained from a single blood donor at the Japanese Red Cross Society (Tokyo, Japan). UT7/Epo-S1 cells (5 × 10^5^) were suspended in 40 μL of the neutralization reaction mixture and allowed to undergo adsorption at 4°C for 2 h. The cells were then transferred to 6-well plates containing 2 mL of RPMI medium supplemented with erythropoietin (2 units/ml) and cultured for 72 h. After incubation, cells were harvested and prepared for fluorescence immunostaining. Infected UT7/Epo-S1 cells were washed once with PBS, followed by treatment with a solution containing 10% FBS, 0.1% Triton X-100, and anti-VP antibody (rabbit polyclonal, 5.5 mg/ml, diluted 1:2000 in PBS) at 25°C for 1 h. The cells were washed three times with PBS and subsequently incubated with a fluorescent-labeled secondary antibody (diluted 1:1000, anti-rabbit IgG AlexaFluor 594 conjugate, Life Technologies, Carlsbad, CA) for 1 h at 25°C. After another set of three PBS washes, flow cytometry was performed to analyze the number of B19V VP-positive cells. During the analysis, 10,000 cells were assessed and the ratio of fluorescently stained cells was calculated to determine the number of positive cells. In Figure 7C, the y-axis represents the relative number of positive cells, with each circle representing an individual sample. Neutralization assays were performed using sera collected from each mouse.

#### ELISpot assays

For ELISpot assays, we killed the immunized mice 2–3 weeks after the second immunization and harvested the inguinal, popliteal, and lumbar lymph nodes. To make single-cell suspensions, we mashed the lymph nodes on an EASYstrainer with 70-μm pores (Greiner Bio-One Co. Ltd., Tokyo, Japan) using a plunger of a 5-mL syringe. The lymph node cells were cultured in RPMI-1640 medium (Sigma-Aldrich, Co., St Louis, MO) supplemented with 10% FBS (Sigma-Aldrich, Co.), 50 mM β-mercaptoethanol (FUJIFILM Wako Pure Chemical Corporation, Osaka, Japan), and 1% antibiotics (Thermo Fisher Scientific Inc.) at 2 ×10^5^ cells/well in MultiScreen® 96-well plates with hydrophobic Immobilon®-P PVDF membrane (Merck KGaA, Darmstadt, Germany). We stimulated lymph node cells with 50 μg/mL of the S-RBD protein or JEprME nanoparticles at 37°C with 5% CO_2_ overnight. Using ELISpot kits (Mabtech AB, Nacka Strand, Sweden), we visualized the spots of IFN-γ- and IL-4-producing cells and counted the number of spots under a light microscope, according to the manufacturer’s instructions.

#### Histology

We killed the immunized mice 2–3 weeks after the second inoculation, perfused the mice with PBS, followed by a 4% paraformaldehyde solution in PBS, and harvested the right quadriceps muscle. The muscles were divided into two segments and embedded in paraffin. We made 4-μm-thick tissue sections using the Retoratome REM-710 (Yamato Kohki Industrial Co., Ltd., Saitama, Japan) and stained the sections with hematoxylin (Sakura Finetek Japan Co., Ltd., Tokyo, Japan) and eosin (Thermo Fisher Scientific Inc.). We semi-quantified the areas of hematoxylin-positive nuclei of infiltrating cells in the muscle to estimate the inflammatory areas using ImageJ (National Institutes of Health MD, Bethesda, USA) with color threshold and measurement tools. Inflammatory areas were expressed as %inflammatory area = [(inflammatory area) / (muscle area)] × 100. To semi-quantify the amount of inoculum remaining in the injected quadriceps muscle, we visualized aluminum, a component of the alum adjuvant that was included in the inoculum of all groups except the PBS group, using lumogallion staining.^49^ Lumogallion, 4-chloro-6-(2,4-dihydoroxypenylazo)-1-phenol-2-sulfonic acid, is a sensitive and selective fluorescent molecular probe for detecting aluminum. Deparaffinized sections were incubated in 1 mM lumogallion (Tokyo Chemical Industry Co., Ltd., Japan) in a 50 mM 1,4-piperazinedielthanesulfonic acid (PIPES) solution (pH 7.4, Sigma Aldrich, USA) for 45 min at 25°C. The sections were washed six times with the PIPES solution and once with distilled water. Sections were counterstained with 4,6’-diamino-2-phenylindole, dihydrochloride solution (DAPI, Dojindo, Kumamoto, Japan), mounted with Dako fluorescent mounting medium (Agilent, California, USA), and observed under a fluorescence microscope (BZ-X810, Keyence, Osaka, Japan). Lumogallion-positive areas were quantified using ImageJ and expressed as %lumogallion area = [(lumogallion-positive area) / (muscle area)] × 100. The mean intensity of the lumogallion-positive areas was calculated as mean intensity = (mean gray value of lumogallion-positive area) - (mean gray value of background). The amount of inoculum remaining in the muscle was estimated using the following equation: aluminum load = %lumogallion area × mean intensity.

### Quantification and statistical analysis

Statistical analysis was performed using Student’s *t*-test or ANOVA and Dunnett’s multiple comparison using GraphPad Prism. The notation of significance on the graph is as follows: ∗∗∗∗ *P* < 0.0001; ∗∗∗ *P* < 0.001; ∗∗ *P* < 0.01; and ∗ *P* < 0.05. The statistical details of all experiments can be found in the legend.
