# Supplementary material for: Flavivirus-based bivalent nanoparticle vaccines induce neutralizing antibodies and Th1 responses against flavivirus and coupling antigens
Source: iScience. 2025 Oct 4;28(11):113659. doi: 10.1016/j.isci.2025.113659 (PMC12589883; doi:10.1016/j.isci.2025.113659)
Supplement: Document S1. Figures S1–S5 [file mmc1.pdf]

iScience, Volume *28*

Supplemental information

Flavivirus-based bivalent nanoparticle vaccines induce neutralizing antibodies and Th1 responses against flavivirus and coupling antigens

Koga Ii, Fumitaka Sato, Yu Hatakeyama, Hidehiko Suzuki, Takafumi Noguchi, Kotaro Ishida, Masashi Arakawa, Kazumasa Nakamura, Ryuta Iwatsuki, Cong Thanh Nguyen, Akiho Yoshida, Nobuyuki Tanaka, Ikuo Tsunoda, Hirotaka Ebina, and Eiji Morita

Supplemental information

Supplemental Figures

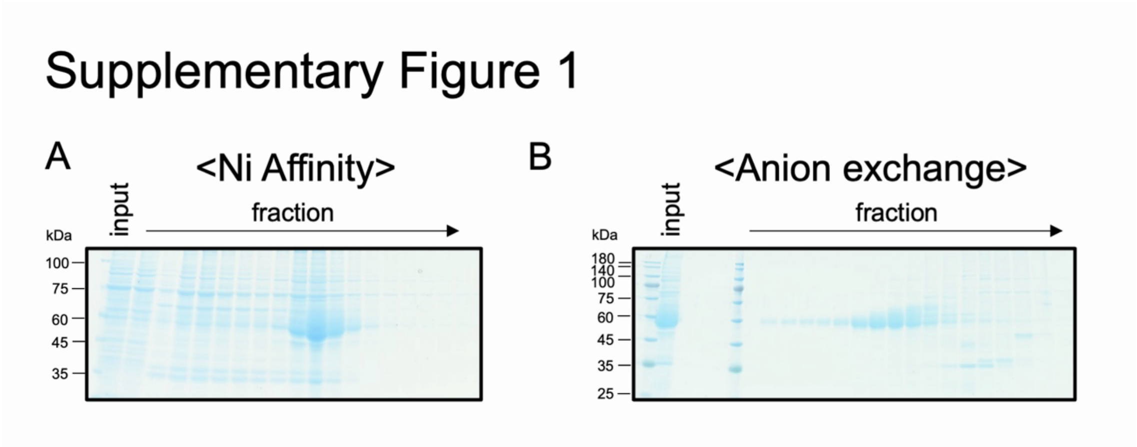

Figure S1. Purification of SpyCatcher-fused SARS-CoV-2 S-RBD proteins. (A) The results of Ni affinity purification. Secreted SpyCatcher-fused SARS-CoV-2 S-RBD proteins were loaded onto a Ni affinity column. After column washing, bound proteins were eluted by an elution buffer [10 mM Tris-HCl (pH 8.0), 400 mM NaCl, 5 mM $MgCl_2$, 10% glycerol, 1 M imidazole] gradient. After separation by SDS-PAGE, the samples were detected by Coomassie brilliant blue (CBB) staining. (B) The results of anion exchange column chromatography purification. After Ni affinity purification, the dialyzed sample was subjected to Q Sepharose Fast Flow IEX column chromatography. Bound proteins were eluted by gradually increasing the salt concentration. After separating by SDS-PAGE, samples were detected via CBB staining.

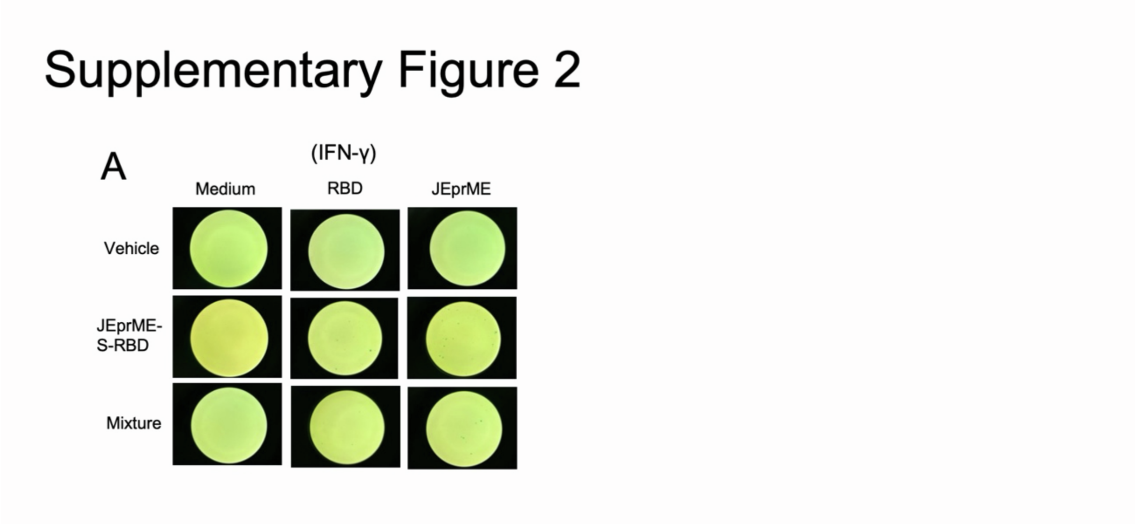

Figure S2. Imaging of ELISpot assays using lymph node cells. The inguinal, popliteal, and lumbar lymph nodes were harvested from the mice at 2–3 weeks after the second inoculation with vehicle (upper panels), JEprME-S-RBD nanoparticles (middle panels), or the mixture antigens (bottom panels). Following stimulation of lymph node cells without (left panels) or with the S-RBD (center panels) or with JEprME (right panels) antigens, we detected IFN-γ-producing cells.

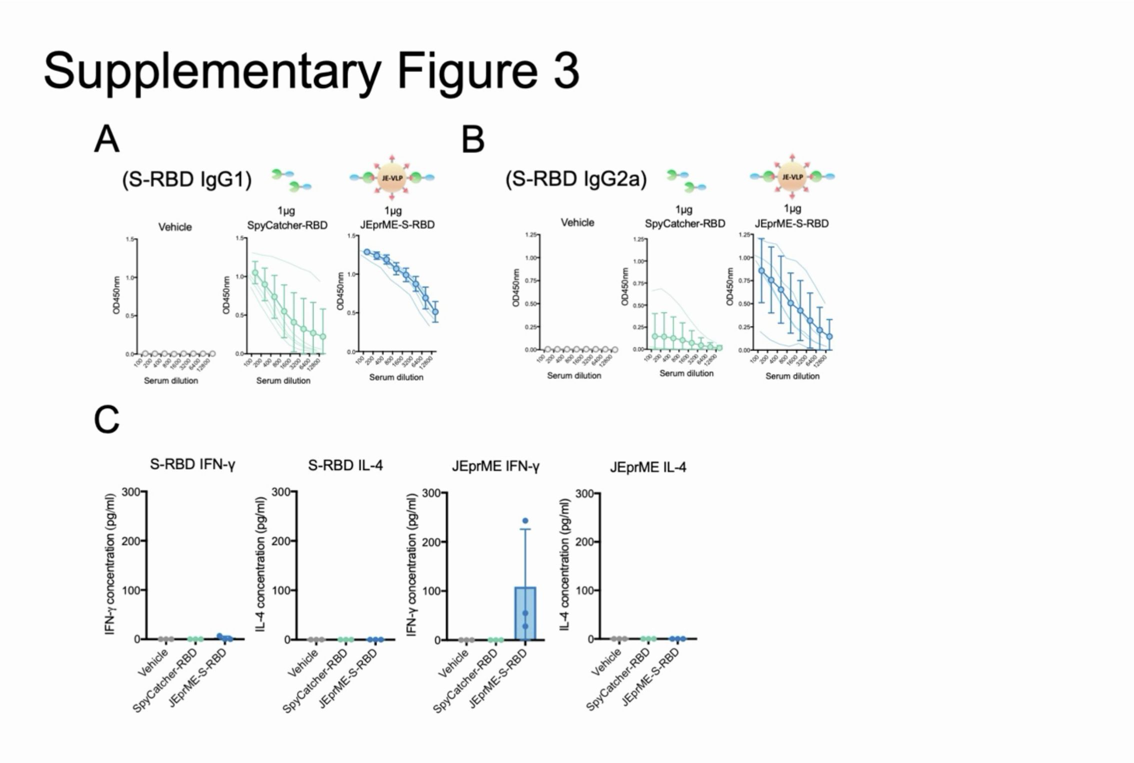

Figure S3. Antibody production and Th1/Th2 responses induced by the immunization of purified SpyCatcher fused S-RBD proteins. (A-B) We titrated SARS-CoV-2 S-RBD-specific IgG1 (A) and IgG2a (B) isotype responses by ELISAs, using sera from mice immunized with SpyCatcher fused S-RBD proteins (SpyCatcher-RBD). Sera from mice that received vehicle or JEprME-S-RBD nanoparticles were used as the negative and positive controls, respectively. Sera were diluted two-fold from a 100-fold dilution. Thin lines represent the IgG isotype levels of individual mice; open circles and bold lines represent the mean IgG isotype levels in each group. Error bars represent standard deviation (n = 5). (C) Concentrations of IFN-γ or IL-4 in the culture supernatants following stimulation with either SARS-CoV-2 S-RBD or JE-prME antigens. Each point represents the cytokine concentrations per well, and bars indicate the mean concentrations for each group (n = 3).

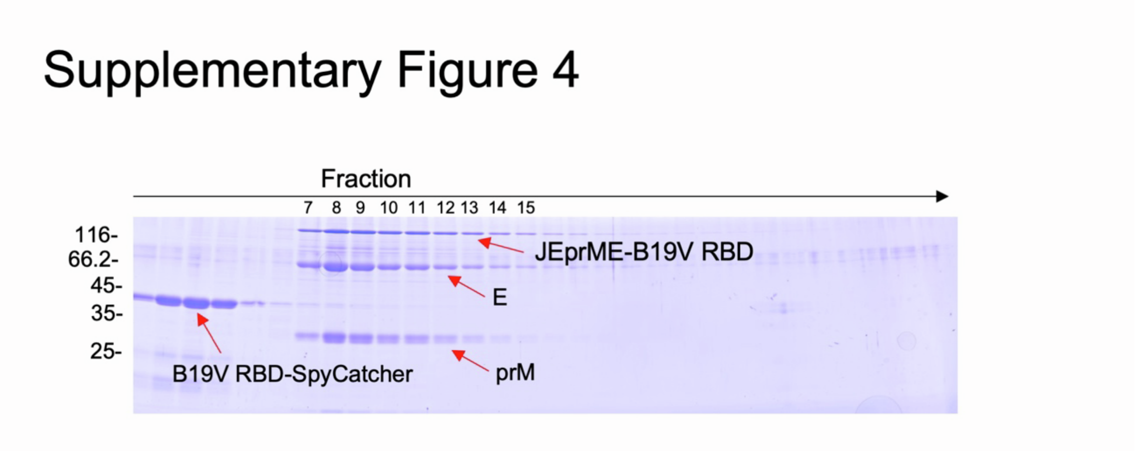

Figure S4. Purification of JEprME-B19V RBD nanoparticles. Reducing SDS-PAGE showing the purification process of JEprME-B19V RBD nanoparticles. After binding of SpyCatcher fused B19V VP1-RBD to the JEprME-SpyTag, protein nanoparticles were fractionated by 15–60% (w/v) sucrose density gradient ultracentrifugation. After separating by SDS-PAGE, samples were detected by CBB staining. Red arrows indicate B19V RBD-SpyCatcher, JEprME-B19V RBD, cleaved E and prM, respectively.

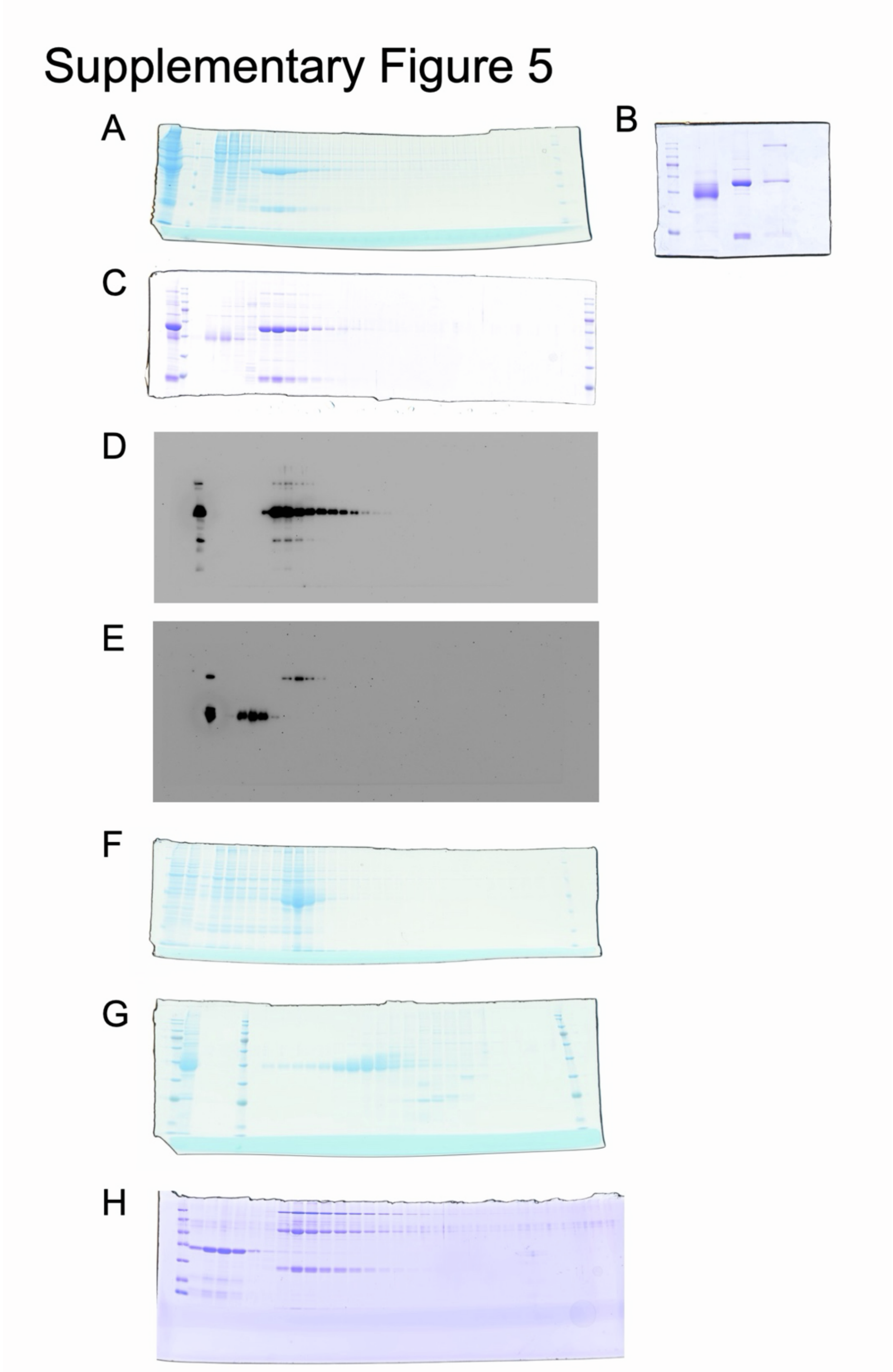

Figure S5. Original western blotting or CBB staining pictures for those presented in main text or supplemental materials. (A) CBB staining gel picture for Figure 3A. (B) CBB staining gel picture for Figure 3D. (C) CBB staining gel picture for Figure 3E. (D) Western blotting picture for Figure 3F. (E) Western blotting picture for Figure 3G. (F) CBB staining gel picture for Figure S1A. (G) CBB staining gel picture for Figure S1B. (H) CBB staining gel picture for Figure S4.
